# Observed deep energetic eddies by seamount wake

**DOI:** 10.1038/srep17416

**Published:** 2015-11-30

**Authors:** Gengxin Chen, Dongxiao Wang, Changming Dong, Tingting Zu, Huijie Xue, Yeqiang Shu, Xiaoqing Chu, Yiquan Qi, Hui Chen

**Affiliations:** 1 State Key Laboratory of Tropical Oceanography, South China Sea Institute of Oceanology, Chinese Academy of Sciences, Guangzhou, China; 2 Marine Science College, University of Information Science and Technology, Nanjing, China; 3 Department of Atmospheric and Oceanic Sciences, University of California, Los Angeles, USA; 4 School of Marine Sciences, University of Maine, Orono, Maine, USA; 5 Key Laboratory of Tectonics and Petroleum Resources of Ministry of Education, Faculty of Resources, China University of Geosciences, Wuhan, China

## Abstract

Despite numerous surface eddies are observed in the ocean, deep eddies (a type of eddies which have no footprints at the sea surface) are much less reported in the literature due to the scarcity of their observation. In this letter, from recently collected current and temperature data by mooring arrays, a deep energetic and baroclinic eddy is detected in the northwestern South China Sea (SCS) with its intensity, size, polarity and structure being characterized. It remarkably deepens isotherm at deep layers by the amplitude of ~120 m and induces a maximal velocity amplitude about 0.18 m/s, which is far larger than the median velocity (0.02 m/s). The deep eddy is generated in a wake when a steering flow in the upper layer passes a seamount, induced by a surface cyclonic eddy. More observations suggest that the deep eddy should not be an episode in the area. Deep eddies significantly increase the velocity intensity and enhance the mixing in the deep ocean, also have potential implication for deep-sea sediments transport.

Surface eddies are frequently observed in the Ocean and have substantial impact on physical and biogeochemical budget^1^,^2^,^3^,^4^,^5^,^6^. However, due to lack of observation, deep eddies are still poorly understood and less studies focused on the phenomena^7^,^8^,^9^,^10^,^11^,^12^. The South China Sea (SCS) is known for the presence of strong eddy activity^13^. The northern SCS has a broad continental shelf and a steep continental slope (Fig. 1a) with numerous seamounts. A strong boundary current exists in the northern SCS and flows southward near the Xisha area^14^. Surface mesoscale eddies are frequently observed near the Xisha Islands, including eddies locally generated and propagated from the northeastern and eastern regions as well^15^. A strong interaction among currents, eddies and topography is expected in the area.

Samples collected by sediment trap at 1500 m of a mooring in Xisha Trough demonstrate that total particle flux increases abruptly from May 2012, which clearly differentiates from its general fluctuation^16^. Current and temperature observations from the same mooring suggest that one-order larger velocity amplitude than usual and obvious positive temperature anomaly occur at deep layer in May 2012. Through analysis of long-term moored records, a deep energetic and baroclinic eddy is revealed. The deep eddy induces larger velocities in deep layer, and should greatly contribute to deep-sea sediments transport. The deep eddy is generated in a wake when a steering flow in the upper layer passes a seamount, which distinguishes from surface eddy genesis by a stratified flow passing an island. Detailed examination of the deep eddy and other possible deep eddy events are presented in this study, which will add to our knowledge of eddy activities in the SCS and carry implications for future studies of deep eddies in the SCS as well as in other regions.

## Results

### An observed deep eddy

**Figure 1:**
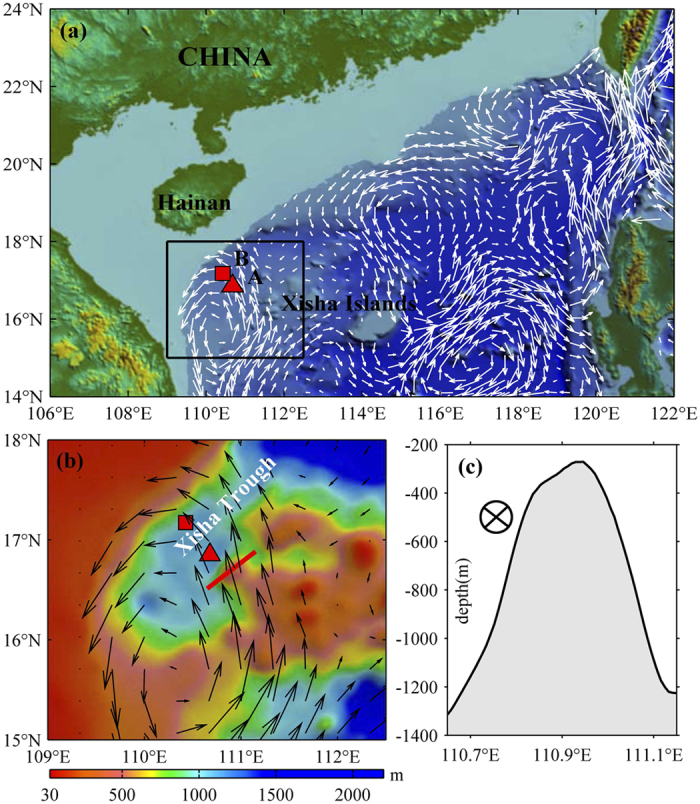
The bathymetry of the northern SCS. **(a)** Colors show the bathymetry of the northern SCS. Triangle and square are locations of Moorings A and B, respectively. The vectors are geostrophic currents on 18 April 2012 from altimeter data. The altimetry data over the shelf shallower than 100 m are masked. **(b)** The topographic map from the bathymetry (color) in Xisha area marked by box in **(a)**. **(c)** The water depth profile along the section marked by red line in **(b)**. Maps are generated using Global Mapper v12.

**Figure 2:**
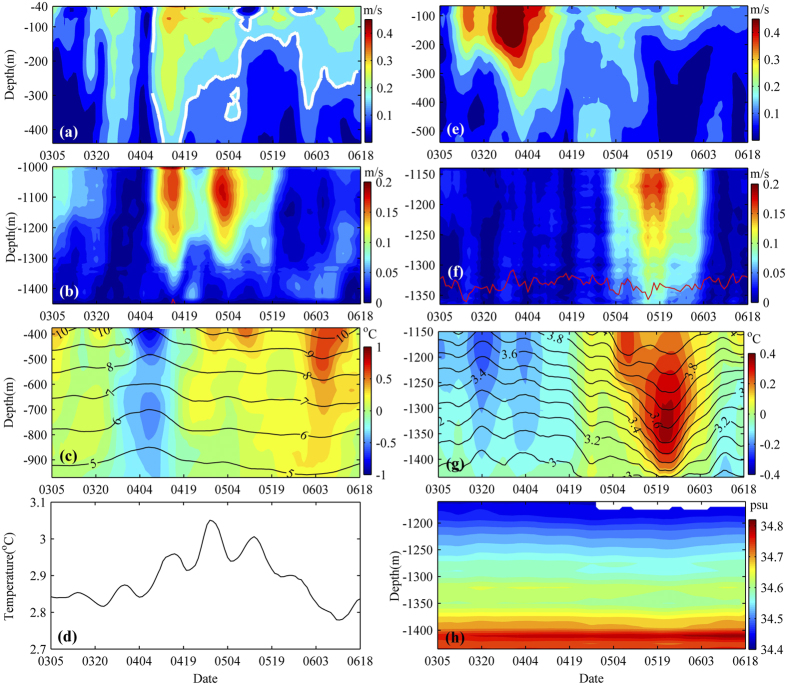
Current and hydrographic observations. Time series of **(a)** 40–440 m and **(b)** 1000–1450 m horizontal velocity magnitude observed by mooring A from 5 Mar 2012 to 18 June 2012. Time series of **(c)** temperature (lines) and temperature anomaly (colors) and **(d)** temperature at around 1463 m from 5 Mar 2012 to 18 June 2012. **(e–g)** Similar as **(a–c)** but observed by mooring B at different depths. **(h)** Salinity time series at 1160–1435 m observed by mooring B from 5 Mar 2012 to 18 June 2012. White lines in **(a)** represent 0.15 m/s contour. Red lines in **(b,f)** represent 75% lines of good data obtained from ADCPs outputs. The data in the layers shallower than red lines have more than 75% good quality with values. Note that red line in **(b)** can only be observed around April 14, 2012 at about 1450 m. In this figure, different colorbars are used. Figures are plotted using MATLAB.

Moorings A and B are deployed adjacent to Xisha Trough at approximately water depth of 1700 m and 1550 m, respectively (Fig. 1; see Method for detail). The velocity amplitudes in the deeper layer (larger than 1000 m) observed at moorings A and B are less than 0.02 m/s for most of the time (Fig. 2b,f). An interesting event is that two maximum velocities with the amplitude of 0.18 m/s occur between 4 April and 8 May 2012 at mooring A. Larger velocity amplitude in the deeper layer can also be observed in Mooring B (Fig. 2f) in May 2012. Observed by sediment trap at 1500 m at mooring B, total particle flux increases abruptly from 179 mg/m^2^/d in May to 398 mg/m^2^/d in July 2012, which presents obvious different fluctuation with that during the same period in 2010 and 2011^16^. It has been reported that strong surface eddies in the SCS can extend vertically to thousands of meters, and thus induce larger velocity in deep layer^17^ and transport sediments^6^. What contributes to the larger velocity amplitude and sediments increase in deep layer shown by the mooring arrays?

An eddy is assumed to consist of solid body rotation within a core (*r* < *R*) and 1/*r* decay elsewhere^7^,^18^. The azimuthal velocity is governed by


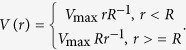


Here *R* is the eddy radius; *r* is the radial distance; and *V*_max_ is the maximum azimuthal velocity. When an eddy passes a mooring, the mooring can record two local maximums of velocity amplitude if an eddy core (*r* < *R*) passes through it, whereas only one maximum of velocity amplitude is recorded in other situations.

Two local maximums of velocity amplitude are shown in the time series of velocity amplitude at mooring A in the 40–440 m layer: the one on 26 March 2012 and another on 14 April 2012 (Fig. 2a). In between a minimum velocity amplitude is recorded on 6 April 2012. The SLA and geostrophic currents suggest that the mooring is passed by a surface, southwestward-propagating, cyclonic eddy at that time (Supplementary Figure S1; Fig. 1a,b). The time when the smallest velocity occurred on 6 April 2012 (Fig. 2a) is when the eddy center passes the mooring. The anomalous temperature series with respect to the one-year time-averaged temperature (colors in Fig. 2c) suggests that the surface cyclonic eddy’s impact could extend to deeper than 970 m. Mooring B recorded larger velocity in the upper layer but with only one velocity core (Fig. 2e). The obvious discrepancy recorded by the two nearby moorings could be attributed to the eddy moving in different trajectories across the observational moorings, and impact of the SCS western boundary current.

**Figure 3:**
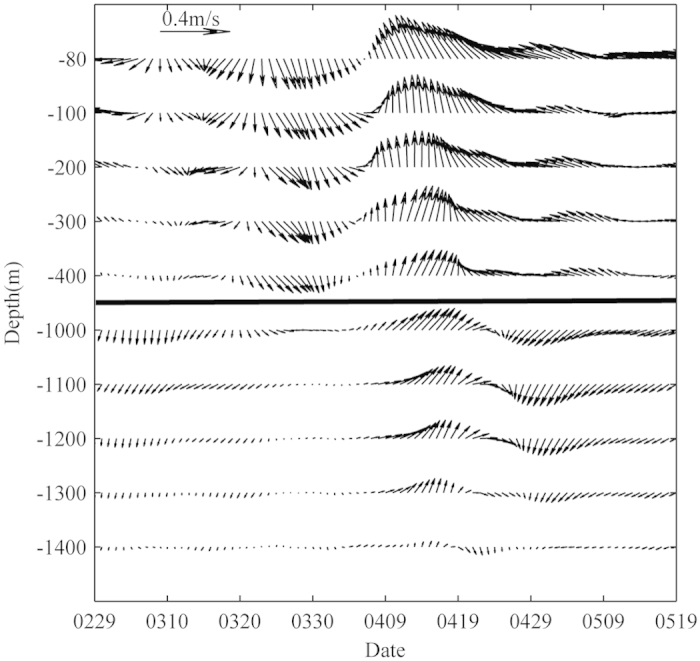
Observed current vectors. Time series of velocities observed by mooring A at different depths from 29 Feb 2012 to 19 May 2012. Figure is plotted using MATLAB.

Structure of larger velocity amplitude in the deeper layer is obviously asynchronous from that of the upper water column. For example, at mooring A, a velocity core with a local maximum of 0.18 m/s at 1100 m occurs on May 5 (Fig. 2b), while the maximum velocity amplitude is only 0.13 m/s at 400–440 m and no obvious velocity core is observed at 200–440 m at that time (Fig. 2a). Larger velocity amplitude with the value of 0.15 m/s (the white line in Fig. 2a) is observed in the upper 460 m layer before April 19. However, the isoline of 0.15 m/s gradually rises to 150 m on May 19. The discrepancy of the upper and deep layers between March 20 and May 9, 2012 at mooring A can be better viewed in velocity vectors (Fig. 3). Because of the effect of the surface cyclonic eddy, the counterclockwise rotation is presented in the upper 400 m layer. Different from the situation in the upper layer, very weak velocities are observed in the layer below 1000 m before April 9. After April 9, the water deeper than 1000 m spins clockwise. These results suggest that the local maximum of the current speed at 1100 m shown in Fig. 2b is not an extension of the surface cyclonic eddy. The highest temperature at 1460 m between March and June appears on April 25, 2012 with the value of 3.05 °C (Fig. 2d), 0.16 degree warmer than the mean temperature at the depth. Note that April 25 is also the middle time of the two maximum velocity amplitudes observed by mooring A in the deeper layer (Fig. 2b).

At mooring B, larger velocities are clearly seen between May 4 and June 3 and the average at 1140–1300 m reaches 0.10 m/s (Fig. 2f). In contrast to the larger velocity in deeper layer, the mean velocity amplitude in the upper layer (500–540 m) is only 0.06 m/s in the same period (Fig. 2e). Within one month around May 22, the isotherm at 1150–1440 m observed by mooring B (Fig. 2g) considerably deepens. Taking the 3.6 °C isotherm as an example, the depth is 1220 m on April 19, but deepens to 1340 m on May 20, and then comes back to 1240 m on June 18. The amplitude of the isotherm reaches ~120 m. A warm core with the temperature anomaly of 0.37 °C can be found at 1200–1400 m (color in Fig. 2g). The salinity changes little (Fig. 2h), but a low-salinity core (~−0.03 pus) can also be identified at about 1350 m around May 19.

Surface eddies in the SCS deepen or raise the isotherms dramatically in the thermocline (~60 m) where a strong stratification is observed^15^, but they have little impact on the isotherms in deep ocean where the stratification is relatively weaker than that in the thermocline. However, when an eddy occurs in deep ocean, the situation is different. Because of weaker density vertical gradient in deep ocean, the same intensity of the perturbation could lead to more dramatic vertical movement of isotherms.

The above analysis suggests that an event appears at deep layer in the SCS with high temperature anomaly and large velocity amplitude (one order larger than the background velocity). The event deepens isotherm in deep layer up to 120 m, spins clockwise, and lasts for one month. Besides, the event is not extension of a surface eddy and so on. It should be a deep eddy that affects the water columns at mooring A and then mooring B.

Now we estimate the eddy radius *R* according to our observations. Firstly, we identify the eddy translation direction past the mooring. The direction is perpendicular to the vector difference *V*_1,max_ − *V*_2,max_^19^. Here, *V*_1,max_ and *V*_2,max_ correspond to the velocities at the two time points *T*_1,max_ and *T*_2,max_ when the eddy core *R* crosses the mooring. Secondly, the translation velocity *U* is estimated by thermal wind balance
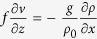
20. Here, *f* is Coriolis frequency, *ρ* is the potential density and *ρ*_0_ is the average potential density computed from the average potential density at 1160–1435 m over the time series (*ρ*_0_ = 1027.3 kg/m^3^). 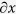 is estimated as Δ*x* = *−U*Δ*t*, where Δ*t* is the time interval of the maximum velocity and the minimum velocity induced by the eddy. *v* is the velocity component perpendicular to the direction of *x*. Finally, based on the eddy translation direction, the vector directions of *V*_1,max_ and *V*_2,max_ and the distance between the two points at *T*_1,max_ and *T*_2,max_, we have an estimate of *R* by solving a trigonometric function. Using the velocity at 1150–1300 m observed by mooring A, we find the eddy propagates westward with an angle of 156 ± 4° from east. That’s why the deep eddy is observed later by mooring B. According to the temperature and salinity measured by mooring B, the eddy’s northwestward-propagating velocity is estimated as 1.27 ± 0.45 cm/s. Thus, the mean radius *R* of the deep eddy at 1150–1300 m is estimated as 21.3 ± 7.6 km. The notion of having a deep eddy with its core area first passing through mooring A followed by its fringe passing through B is supported by the kinematic analysis above. The characteristic Rossby number of the eddy can be estimated through the relative vorticity magnitude and the Coriolis coefficient as *R*_0_ = 2*V*_max_/*Rf.* The anticyclonic eddy has a finite Rossby number of 0.39, which is in the same scale of the two coherent eddies affecting deep water in the Labrador Sea^18^.

Because of the larger eddy rotation speed and the smaller eddy size, maybe we couldn’t ignore eddy rotation to estimate the eddy radius. If eddy rotation is considered, the relationship will be 
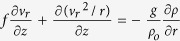
 by assuming that eddy rotation speed *ν*_*r*_ is far larger than its propagation velocity. The formula can be wrote as 
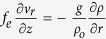
, where the effective vorticity *f*_*e*_ = *f* + *w* and angular velocity 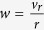. Because of *w* < 0 for an anticyclonic eddy, the effective vorticity *f*_*e*_ should be smaller than the situation that eddy rotation is not considered. This means the eddy size estimated using thermal wind balance is smaller than its realistic size. Further discussion can be found in the following text.

### Eddy generation

Surface eddies can be generated when a stratified flow passes an island, that is a common phenomenon in the ocean^21^,^22^,^23^. A flow passing a seamount could also be accompanied with eddy genesis, not in the upper layer but in the deep layer. Our observation suggests that a surface cyclonic eddy in Xisha area induces a strong and long-lasting current (Figs 1b, 2b, 3 and S1) in April 2012. We propose a possible scenario as follows. When the vertically-shearing current flows over a seamount (Fig. 1b,c), the friction between the current and the seamount destroys the stratification near the bottom and induces a bottom mixing layer or a bottom boundary layer, i.e., a layer with vertically uniform density, which results in a horizontal density front. Accompanying the horizontal front is a frontal jet subject to the geostrophic constrain. The instability of the frontal jet can lead to a coherent eddy structure, especially an anticyclonic eddy when the branch flow of the surface eddy passes the seamount on its left side. That’s why stronger deep currents are observed after April 9 (Fig. 3). Compared with eddies in the upper layer induced by flow passing an island, eddies related to seamount should be stronger in deep layer.

**Figure 4:**
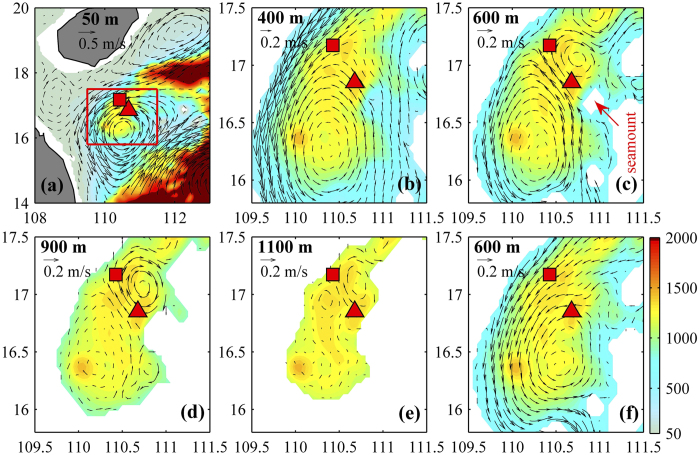
Simulated currents at different depths. **(a–e)** Modeled current vectors at 50 m, 400 m, 600 m, 900 m and 1100 m. **(f)** Same as **(e)** but for the situation that the seamount shown in Fig. 1c is removed. Triangle and square represent the locations of the moorings A and B, respectively. The region in **(b–f)** is marked by red box in **(a)**. Color shows the bathymetry, and white means the land. Maps are generated using MATLAB.

A South China Sea model (see Method for the analysis) is utilized to further the investigation of the effects of the seamount on the deep eddy qualitatively. The seamount reproduced by the model can be clearly identified although it is not exactly the same as that shown in Fig. 1b,c, because of the smoothed topography and model resolution. The model can reproduce the surface cyclonic eddy and the deep eddy as observed (Fig. 4). The cyclonic eddy at 400 m (the height of the seamount in the model) and the above can be seen clearly (Fig. 4a,b). At 600 m and deeper layer, the effect of seamount is evident: an anticyclonic eddy is generated to its north (Fig. 4c–e). When the seamount is removed by interpolating the surrounding depth to the seamount area, no anticyclonic deep eddy is found (Fig. 4f), which confirms the generation mechanism of the deep eddy as a steering flow passing a seamount.

**Figure 5:**
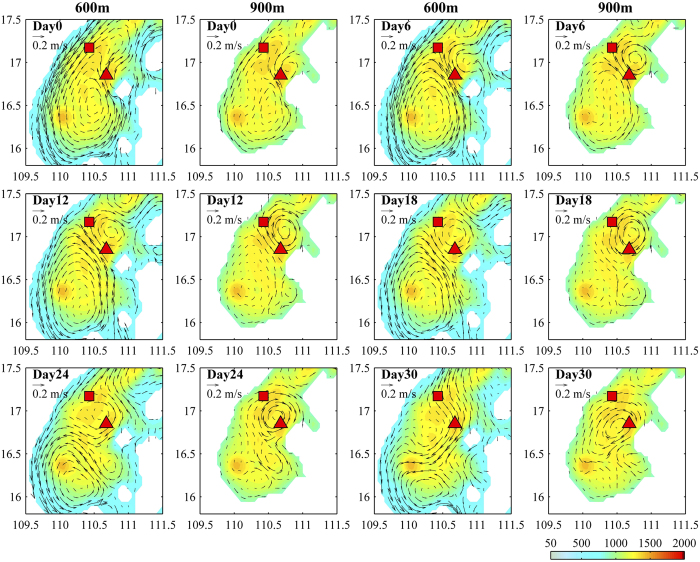
Evolution of the simulated deep eddy. Evolution of the simulated deep eddy shown in Fig. 4 at 600 m and 900 m. Color shows the bathymetry, and white means the land. Maps are generated using MATLAB.

The model outputs are used to further understand the deep eddy evolution. Because the northward branch flow of the surface eddy passes the seamount on its left side (Day 0 in Fig. 5), the anticyclonic deep eddy rather than a cyclonic eddy is generated to the north of the seamount. The deep eddy becomes stronger and extends northwestward (Days 6 and 12), and then propagates westward due to β effect when the surface eddy leaves and surface current becomes weak (Days 12, 18 and 24). Due to the restriction of topography, the deep eddy finally prorogates southwestward along the isobaths (Days 24 and 30).

Compared with at 600 m, the deep eddy tends to be stronger at 900 m (Fig. 5). For details, the deep eddy can be identified at 900 m but not at 600 m on Days 0 and 6, is stronger at 900 m than at 600 m on Days 12, 18 and 24, and disappears at 600 m but still exists at 900 m on Day 30. These results suggest the deep eddy is generated in deep layer, which implicates that only a seamount with enough size could induce a robust Taylor vortex. Thus, the energetic (maximum-speed) depth of a deep eddy should be determined by the combined effect of steering flow strength and seamount size.

Eddy size is controlled by the baroclinic deformation radius *R*_*d*_ and the seamount size *D*^21^. When *D* is bigger than *R*_*d*_, the eddy size is controlled by *R*_*d*_, otherwise the eddy size is approximately the same as *D*. For the particular seamount of consideration, *D* is about 46 (56) km at a depth of 1000 (1200) m. Based on the WOA09 data, the mean Brunt–Väisälä frequency *N* at the mooring locations in the upper 1000 (1200) m is 0.0149 (0.0149) s^−1^ in April. If we set the vertical scale *H* as 1000 (1200) m and the Coriolis parameter *f* as 4.3 × 10^−5^ s^−1^, the *R*_*d*_ can be estimated by *R*_*d*_ = *NH/f *≈ 347 (416) km. This means the eddy size should be controlled by the seamount size, and thus its radius should be on the order of 23 ~ 28 km, which is a bit larger than that estimated using thermal wind balance.

## Discussion

This study has showcased the first observation of a SCS deep eddy. The eddy induces the maximum velocities during the observational period in deep layer with the amplitude of 0.18 m/s, which is far larger than the mean value of 3.4 cm/s. The deep eddy considerably deepens isotherms with the amplitude of ~120 m, suggesting the eddy has an important impact on deep-sea conditions. Based on observation by mooring B, the mean Richardson number 
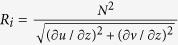
 during April 5 to May 4 2012 at 1160–1320 m, where good-quality temperature, salinity and velocity data are available, is 0.0309 whereas it is 0.0219 from May 5 to June 4. The smaller value of *R*_i_ suggests that the deep eddy mentioned above weakens the ocean stratification and intensifies the vertical shear, and thus could induce stronger turbulent mixing. A rough estimate demonstrates that the strong deep eddy event accounts for 24% of the total kinetic energy in deep layer (1175–1220 m) over the three-year observation period from May 2009 to August 2012 by mooring B (Table 1). Strong current and mixing induced by the deep eddy should lead to the abruptly increase of the particle flux in Xisha Trough from May 2012^16^.

**Figure 6:**
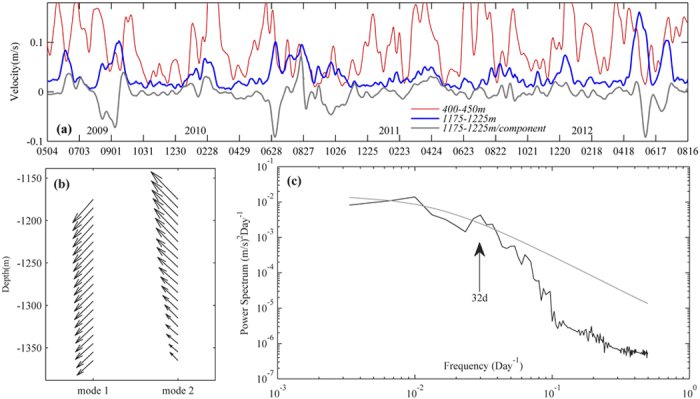
Long-term moored current record. **(a)** Time series of the mean 400–450 m (thin-red line), 1175–1225 m (thick-blue line) velocity amplitudes and the mean across-isobath velocity component at 1175–1225 m (thick-gray line; negative values mean northwestward) observed by mooring B from May 2009 to August 2012. **(b)** Spatial structure of the first two horizontal velocity modes. **(c)** The power spectrum of the mean across-isobath velocity component at 1175–1225 m and the 95% confidence curve (gray line). Figures are plotted using MATLAB.

The time evolution of 1175–1225 m depth-averaged velocities (blue line in Fig. 6a) suggests more than 72% of velocities are less than 0.04 m/s during May 2009 and August 2012. However, larger velocities, even larger than that at 400–450 m (red line in Fig. 6a), are also observed, for example, in June 2009, September 2009, August 2010, May 2012 and so on. These events last longer than 3 weeks and all of them correspond to strong northward currents at earlier time in the upper layer (Figure S2). Empirical orthogonal function analysis is applied to analyze the velocity observations by mooring B. The result shows that the first three dominant modes account for 68%, 16% and 6%, respectively, of the total variance. Mode 1 demonstrates the deep currents are mainly southwestward or northeastward (Fig. 6b), which should be attributed to the impact of the west boundary current in the SCS. Besides, the deep currents are nearly uniform as the no eddy situation in most of the time shown in Fig. 2f. Mode 2 represents the sheared deep currents, implying that some events affect the deep-sea conditions. The sheared currents tend to be across-isobath slanted in the northwest-southeast direction.

The power spectrum of the across-isobath velocity component (gray line in Fig. 6a negative values mean northwestward) shows a significant peak centered at a period of 32 d (Fig. 6c). If we set island size *D* as 50 km, unperturbed upstream velocity *U* as 0.1 m/s and eddy shedding interval *T* as 30 d, the Strouhal number *St = D/TU* is estimated to be 0.19. For numerical experiments of fluid around an obstacle^21^ and in a nonrotating frame^24^, the *St* values are 0.23 and 0.2, respectively. The consistence in *St* number with other eddy shedding processes is a further evidence for the present argument that the deep high moment energy events are deep eddies. The significantly increased velocity amplitudes, kinetic energy and the cross-slope currents in deep layers could be attributed to deep eddies, which is thus not a negligible factor when the SCS deep circulation and deep-sea sediments are examined. More observations are desirable to further understand deep eddies and their regional impact.

Based on the numerical model, another two deep eddies (E1 and E2) in different situations are shown (Figure S3). In the E1 case, no strong surface current exists in the upper 600 m near the seamount. However, due to topographic steering, a cyclonic circulation and thus a northward current appear at 900 m near the seamount. As a result, the deep eddy E1 appears at 900 m but not at 600 m during its whole life. E1 is relatively weak and tends to be local. In the E2 case, a strong cyclonic circulation with a large northward current appears near the seamount. Besides, a strong northeastward current exists to the north of 17°N. The energetic E2 finally propagates northeastward along the isobaths due to the effect of background flow. These cases indicate that the Xisha Trough is an area of deep eddy generation, and deep eddies can dissipate locally or propagate in different directions due to β effect, background currents and so on.

## Methods

### Measurements from the mooring system

The primary measurements are obtained from two moorings (A and B in Fig. 1). Mooring A was deployed approximately at 16.85°N, 110.68°E in the northwestern SCS (A in Fig. 1) from August 2011 to August 2012, containing an upward-looking 75 kHz and a downward-looking 150 kHz Workhorse Acoustic Doppler Current Profiles (ADCP) at depths of about 465 m and 972 m, respectively. Between the two ADCPs, 11 Temperature Logger Sea-Bird Electronic (SBE) 56s at a uniform interval of 50 m, measure single-point temperature series. In addition, a SBE 56 and a SBE 37 at about 370 m record a single-point temperature and pressure series. In addition, another SBE 37 measures temperature at about 1463 m.

Mooring B was deployed at approximately at a location 17.17°N, 110.43°E on May 2009. This mooring also had an upward-looking 75 kHz and a downward-looking 150 kHz ADCPs. From August 23 2011, 21 Infinity conductivity and temperature sensors (CT), which are data logger for temperature and salinity measurements, were added in the mooring below the 150 kHz ADCP at 15 m interval, and a Seaguard Recording Current Meter (RCM), which records a single-point temperature, pressure and current, was set at the bottom of the mooring. The RCM could be used to assist in inferring the depth of CT and to amend the measurement error of the downward-looking ADCP at the sea floor.

Vertical resolution of the upper and lower ADCPs is 8 or 16 m and 4 m, respectively. Sampling time frequency of the ADCP is every 30 min or 1 h. The time interval of CT and the RCM is 1 h, whereas the time interval is 10 min for the two SBE 37s and 30 s for SBE 56s. In this study, the ADCP current velocities, the temperate and salinity measured by Infinity-CTs and SBE 56s are linearly interpolated onto a uniform 5 m interval. Time series of the measurements are averaged to daily intervals. More details of the mooring measurements used in this study are summarized in Table 1.

### Sea surface data from satellites

Daily geostrophic current and sea surface height anomaly derived from the altimeter data distributed by the Archiving, Validation, and Interpretation of Satellite Oceanographic data (AVISO) are used to examine mesoscale eddies that could be identified in the surface layer. The weekly geostrophic current and sea surface height distributed by AVISO are averaged into monthly climatological data to verify the model performance.

### Model setup

The model is configured around the South China Sea (99°E–140°E, 1°N–30°N) by the Regional Ocean Modeling System^25^ (ROMS) with a horizontal resolution of 1/20° × 1/20° and 40 vertical layers by terrain-following s-ordinate^26^. The bottom topography is obtained from ETOPO2 with minimum water depth set equal to 5 m, and it is slightly smoothed to reduce truncation error. The model is forced with climatological monthly atmospheric forcing (wind, short wave radiation, precipitation, 2-m air temperature and humidity, mean sea level pressure, and cloud cover) from ECMWF Re-analysis Interim (ERA-Interim) products (provided by European Centre for Medium-Range Weather Forecasts), surface net heat fluxes are calculated by the Coupled Ocean-Atmosphere Response Experiment (COARE 3.0) algorithm^27^. The model open boundary information is provided by the climatological monthly reanalysis results from Simple Ocean Data Assimilation (SODA). The model is spun up from a rest with initial conditions provided by the climatological temperature and salinity in January from SODA, and it runs for 24 years, the 6-day averaged outputs of the last year are used for analysis.

The model driven by the monthly climatological forcing could reasonably capture features of the seasonal circulation in the SCS (Figure S4), with a basin-wide cyclonic circulation during winter, and a cyclonic northern gyre and an anti–cyclonic southern gyre forming a dipole with a jet streaming away from the coast of Vietnam^28^. The simulated structure of sea surface elevation also agree reasonably well with satellite observation (Figure S4). Note that different colorbars for the observed and simulated sea surface elevations are because the sea surface height in AVISO data is above geiod. The annual mean net volume transport in Luzon strait, an important factor in affecting the SCS circulation^29^, is around 4Sv (1Sv = 10^6^ m^3^/s) westward, and it shows distinct seasonal variability (Figure S5), which agrees well with that summarized by previous study^30^. The good model/data agreements suggest that the model is able to capture the fundamental processing in the SCS, and thus is suitable for the aim of a “process-oriented” study on a steering flow passing a seamount. Besides, the climatological model is beneficial for simplifying the problem and figuring out what actually induces the deep eddy.

We choose a period, during which a cyclonic eddy exists in the upper layer around the Xisha island for more than one month (Fig. 4a), similar to the situation in Mid-April, 2012, to check the velocity patterns in the deeper layers. An experiment run is also performed, in which the seamount is removed by interpolating the surrounding depth to the seamount area.

## Additional Information

**How to cite this article**: Chen, G. *et al.* Observed deep energetic eddies by seamount wake. *Sci. Rep.* **5**, 17416; doi: 10.1038/srep17416 (2015).

## Supplementary Material

Supplementary Information

## Figures and Tables

**Table 1:** Information of the moorings.

	Instrument	Time	Depth (m)	Measurement
Mooring A (16.85°N, 110.68°E) Water depth: 1708 m	ADCP	20110823–20120817	40–440, 1000–1450	u, v
	SBE 56	20110823–20120817	375–970	t
	SBE 37	20110823–20120817	1463	p, t
Mooring B (17.17°N, 110.43°E) Water depth: 1567 m	ADCP	20090504–20100904	35–460, 1170–1430	u, v
	ADCP	20100905–20110822	140–640, 1175–1435	u, v
	ADCP	20110823–20120817	65–540, 1140–1365	u, v
	CT	20110823–20120817	1150–1435 (t), 1160–1435 (s)	t, s

The “Depth (m)” column is denoted as the effective measurements depth.

## References

[b1] Chelton D. B., Gaube P., Schlax M. G., Early J. J. & Samelson R. M. The Influence of Nonlinear Mesoscale Eddies on Near-Surface Oceanic Chlorophyll. Science 334, 328–332, doi: 10.1126/science.1208897 (2011). 21921157

[b2] Chelton D. B., Schlax M. G. & Samelson R. M. Global observations of nonlinear mesoscale eddies. Prog Oceanogr 91, 167–216, doi: 10.1016/j.pocean.2011.01.002 (2011). 21921157

[b3] Dong C. M., McWilliams J. C., Liu Y. & Chen D. K. Global heat and salt transports by eddy movement. Nat Commun 5 3294, doi: 10.1038/Ncomms4294 (2014). 24534770

[b4] Gruber N. *et al.* Eddy-induced reduction of biological production in eastern boundary upwelling systems. Nat Geosci 4, 787–792, doi: 10.1038/Ngeo1273 (2011).

[b5] Mahadevan A. Eddy effects on biogeochemistry. Nature 506, 168–169 (2014). 24476817 10.1038/nature13048

[b6] Zhang Y. W. *et al.* Mesoscale eddies transport deep-sea sediments. Sci Rep-Uk 4 5937, doi: 10.1038/Srep05937 (2014). PMC4120309 25089558

[b7] Carpenter J. R. & Timmermans M. L. Deep mesoscale eddies in the Canada Basin, Arctic Ocean. Geophys Res Lett 39 L20602, doi: 10.1029/2012gl053025 (2012).

[b8] Gordon A. & Greengrove C. Abyssal eddy in the southwest Atlantic. Deep Sea Res. 33, 839–847 (1986).

[b9] Babu M. T., Kumar S. P. & Rao D. P. A Subsurface Cyclonic Eddy in the Bay of Bengal. J Mar Res 49, 403–410, doi: 10.1357/002224091784995846 (1991).

[b10] Chiang T. L. & Qu T. D. Subthermocline Eddies in the Western Equatorial Pacific as Shown by an Eddy-Resolving OGCM. J Phys Oceanogr 43, 1241–1253, doi: Doi 10.1175/Jpo-D-12-0187.1 (2013).

[b11] Zhang Z. X., Qiao F. L. & Guo J. S. Subsurface eddies in the southern South China Sea detected from *in-situ* observation in October 2011. Deep-Sea Res Pt I 87, 30–34, doi: 10.1016/j.dsr.2014.02.004 (2014).

[b12] Drillet Y., Bourdalle-Badie R., Siefridt L. & Le Provost C. Meddies in the Mercator North Atlantic and Mediterranean sea eddy-resolving model. J Geophys Res-Oceans 110, doi: 10.1029/2003jc002170 (2005).

[b13] Wang G. H., Su J. L. & Chu P. C. Mesoscale eddies in the South China Sea observed with altimeter data. Geophys Res Lett 30, doi: 10.1029/2003gl018532 (2003).

[b14] Fang G. H., Wang G., Fang Y. & Fang W. D. A review on the South China Sea western boundary current. Acta Oceanol Sin 31, 1–10, doi: 10.1007/s13131-012-0231-y (2012).

[b15] Chen G. X., Hou Y. J. & Chu X. Q. Mesoscale eddies in the South China Sea: Mean properties, spatiotemporal variability, and impact on thermohaline structure. J Geophys Res-Oceans 116, doi: 10.1029/2010jc006716 (2011).

[b16] Liu J. *et al.* Modern transport and deposition of settling particles in the northern South China Sea:Sediment trape vidence adjacent to Xisha Trough. Deep Sea Res., part I 93, 145–155 (2014).

[b17] Zhang Z., Zhao W., Tian J. & Liang X. A mesoscale eddy pair southwest of Taiwan and its influence on deep circulation. J Geophys Res-Oceans 118, 6479–6494 (2013).

[b18] Lilly J. M. & Rhines P. B. Coherent eddies in the Labrador Sea observed from a mooring. J Phys Oceanogr 32, 585–598, doi: 10.1175/1520-0485 (2002).

[b19] Lilly J. M. *et al.* Observations of the Labrador Sea eddy field. Prog Oceanogr 59, 75–176, doi: 10.1016/j.pocean.2003.08.013 (2003).

[b20] Fan X., Send U., Testor P., Karstensen J. & Lherminier P. Observations of Irminger Sea Anticyclonic Eddies. J Phys Oceanogr 43, 805–823, doi: 10.1175/Jpo-D-11-0155.1 (2013).

[b21] Dong C. M. & McWilliams J. C. A numerical study of island wakes in the Southern California Bight. Cont Shelf Res 27, 1233–1248, doi: 10.1016/j.csr.2007.01.016 (2007).

[b22] Piedeleu M. *et al.* An observational study of oceanic eddy generation mechanisms by tall deep-water islands (Gran Canaria). Geophys Res Lett 36, doi: 10.1029/2008gl037010 (2009).

[b23] Jimenez B., Sangra P. & Mason E. A numerical study of the relative importance of wind and topographic forcing on oceanic eddy shedding by tall, deep water islands. Ocean Model 22, 146–157, doi: 10.1016/j.ocemod.2008.02.004 (2008).

[b24] Zdravkovich M. Flow around Circular Cylinders: A Comprehensive Guide through Flow Phenomena, Experiments, Applications, Mathematical Models, and Computer Simulations. Oxford University Press, 694 (1997).

[b25] Shchepetkin A. F. & McWilliams J. C. The regional oceanic modeling system (ROMS): a split-explicit, free-surface, topography-following-coordinate oceanic model. Ocean Model 9, 347–404, doi: 10.1016/j.ocemod.2004.08.002 (2005).

[b26] Song Y. H. & Haidvogel D. A Semiimplicit Ocean Circulation Model Using a Generalized Topography-Following Coordinate System. J Comput Phys 115, 228–244, doi: 10.1006/jcph.1994.1189 (1994).

[b27] Fairall C. W., Bradley E. F., Rogers D. P., Edson J. B. & Young G. S. Bulk parameterization of air-sea fluxes for Tropical Ocean Global Atmosphere Coupled Ocean Atmosphere Response Experiment. J Geophys Res 101, 3747–3764, doi: 10.1029/95jc03205 (1996).

[b28] Fang W., Fang G., Shi P., Huang Q. & Xie Q. Seasonal structures of upper layer circulation in the southern South China Sea from *in situ* observations. J Geophys Res 107, 3202, doi: 10.1029/2002JC001343 (2002).

[b29] Chen G. & Xue H. Westward intensification in marginal seas. Ocean Dyn 64, 337–345

[b30] Hsin Y, C., Wu C. & Chao S. An updated examination of the Luzon Strait transport. J Geophys Res 117, C03022, doi: 10.1029/2011JC007714 (2012).

