# Supplementary material for: Observed deep energetic eddies by seamount wake
Source: Sci Rep. 2015 Nov 30;5:17416. doi: 10.1038/srep17416 (PMC4663540; doi:10.1038/srep17416)
Supplement: Supplementary Information [file srep17416-s1.pdf]

• **Supplementary information**

Observed deep energetic eddies by seamount wake

Gengxin Chen, Dongxiao Wang, Changming Dong, Tingting Zu, Huijie Xue, Yeqiang Shu, Xiaoqing Chu, Yiquan Qi & Hui Chen

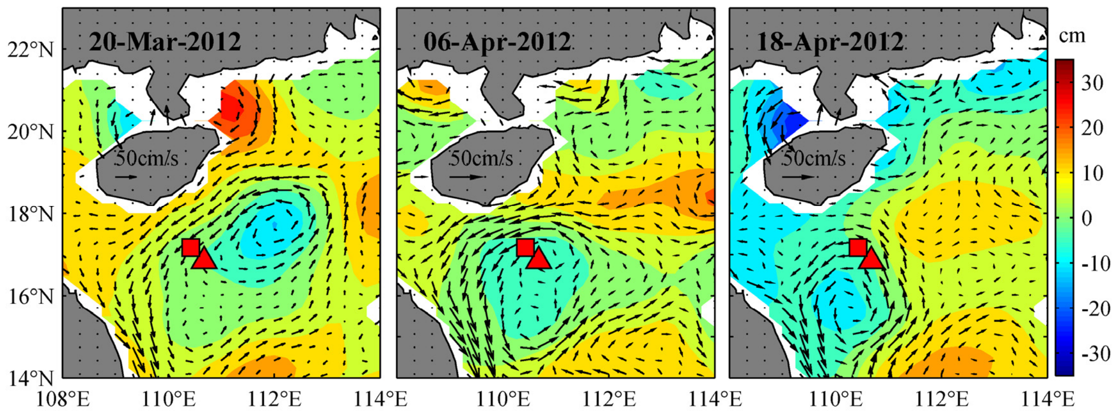


**Figure S1.** Sea level anomalies (colors) and geostrophic currents (vectors) from March through April of 2012. Triangle and square represent the locations of the moorings A and B, respectively. Maps are generated using MATLAB.

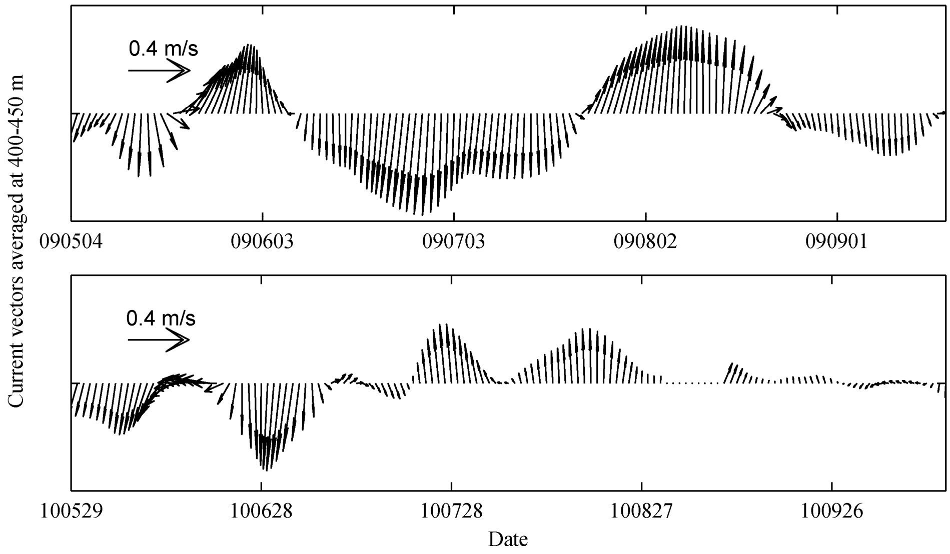


**Figure S2.** Current vectors observed by mooring B averaged at 400-450 m from May to September 2009 and from May 2010 to September 2010. Figures are plotted using MATLAB.

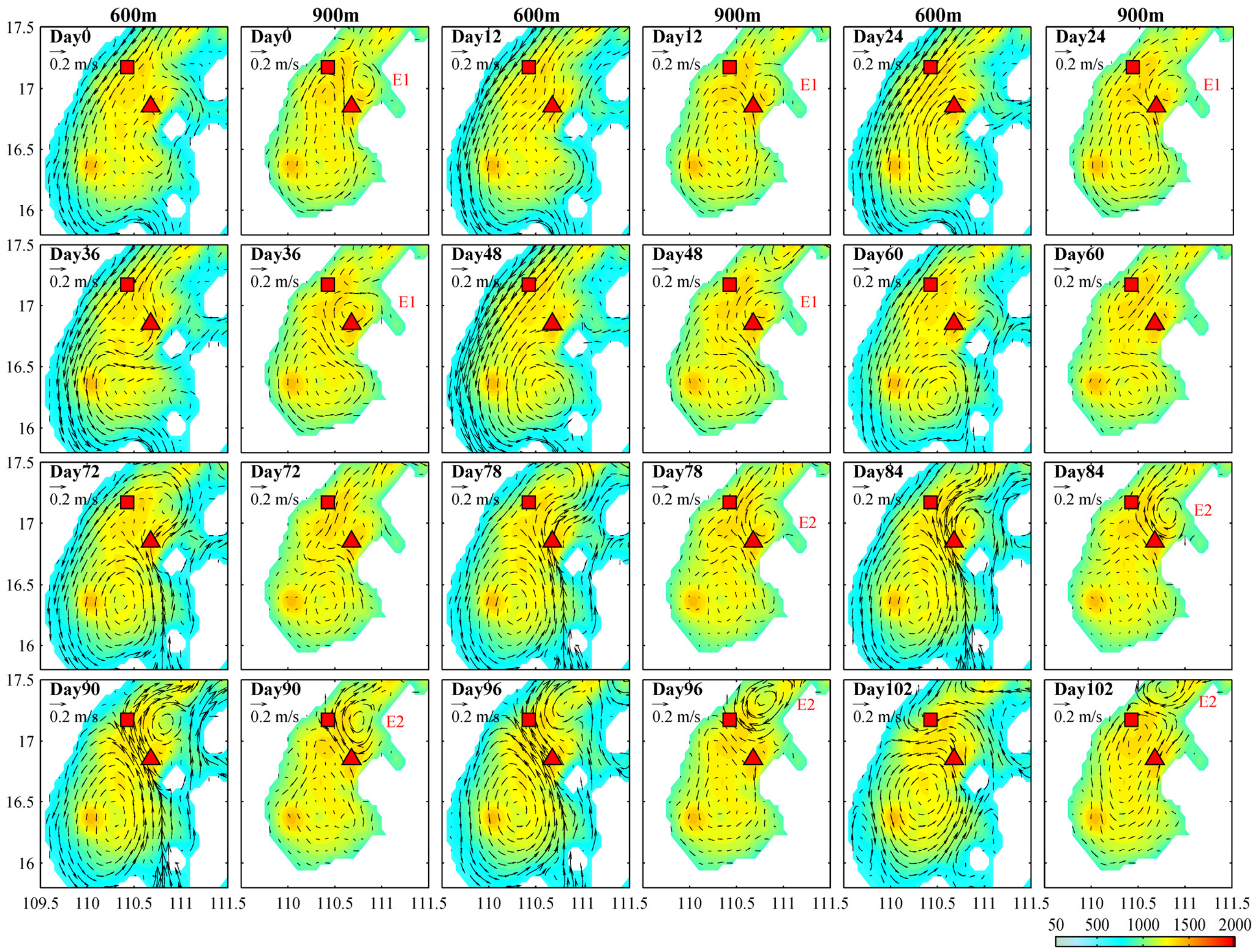


**Figure S3**. Simulated currents at 600 m and 900 m in Xisha area for two cases of deep eddies E1 and E2. In order to keep consistent with Figures 4 and 5, triangle and square representing locations of moorings A and B are also plotted. Maps are generated using MATLAB.

During Days 0-48, no strong surface and northward current exist in the upper 600 m near the seamount. However, due to topographic steering, a cyclonic circulation and thus a northward current appear at 900 m near the seamount. As a result, the deep eddy E1 appears at 900 m but not at 600 m during its whole life. E1 is relatively weak and tends to be local. Subsequently, a strong cyclonic circulation with a large northward current near the seamount appears on Days 72-96. In addition, a strong

northeastward current exists to the north of 17°N from Day 78. E2 is generated on Day 78, grows in size subsequently, and propagates northeastward along the isobaths from Day 90 due to the effect of background flow.

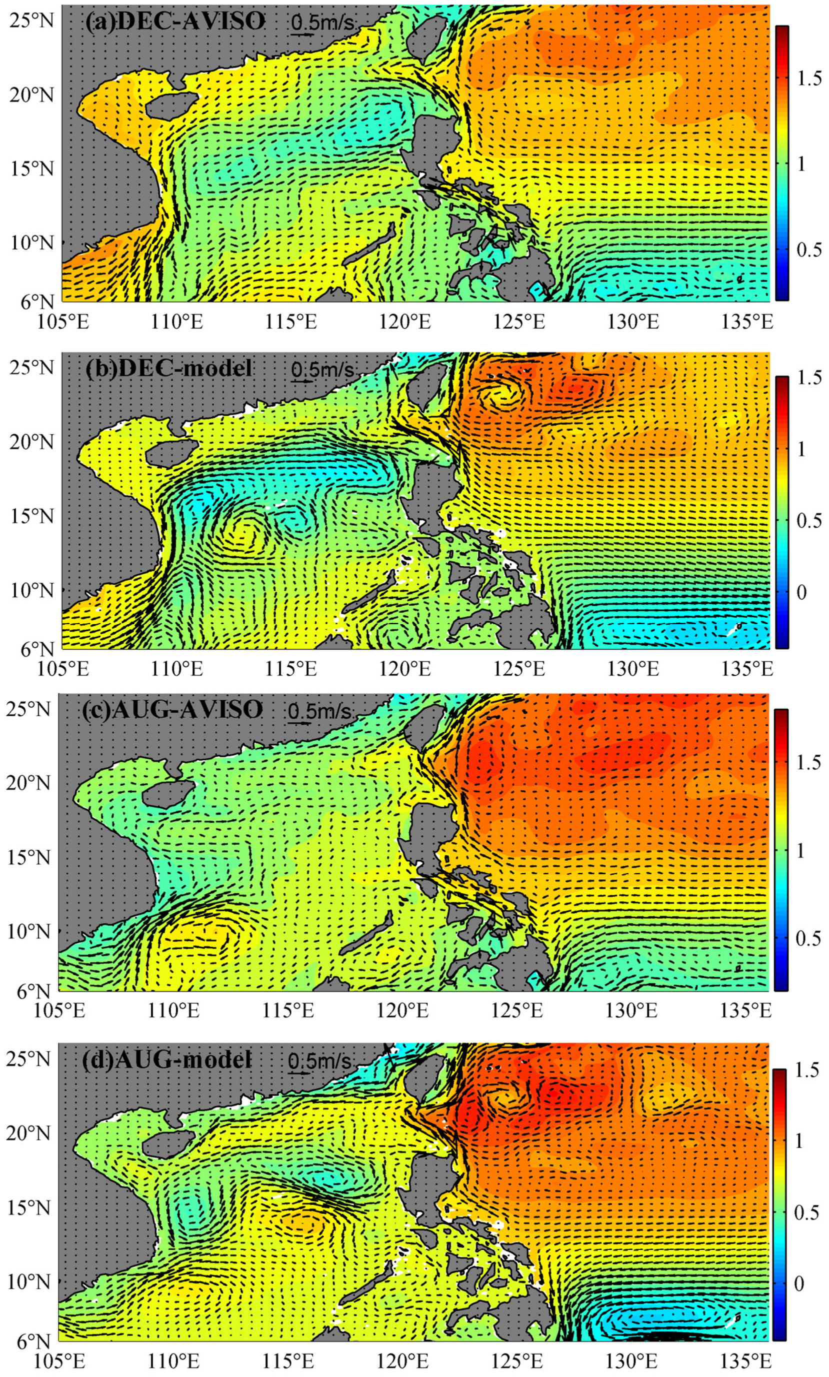


Figure S4. Climatology surface elevations (color; m) and current fields (vector; m/s)

in December for (a) AVISO data averaged from 1993 to 2012, (b) the last 4-year mean fields simulated with monthly climatological forcing. (c-d) Same as (a-b) but for August. Note that different colorbars for the observed and simulated sea surface elevations are because the sea surface height in AVISO data is above geiod. Maps are generated using MATLAB.

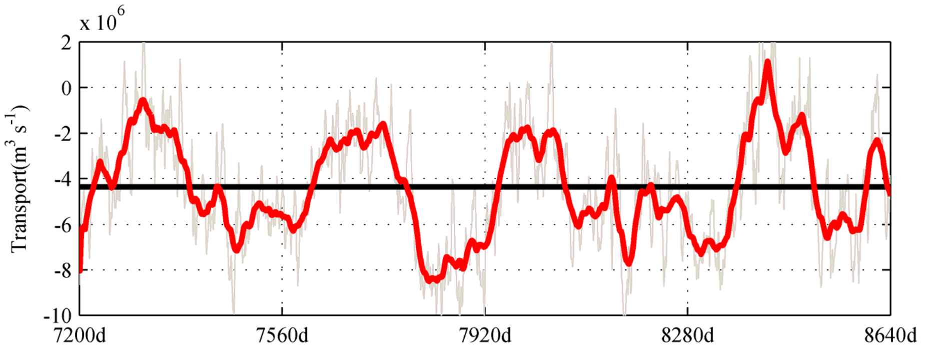


Figure S5. Simulated net volume transports integrated at 120.75°E in Luzon Strait during 7200 days and 8640 days. Gray, red and black lines represent daily, monthly and mean transports. Figure is plotted using MATLAB.
